# The Growing Complexity of UHRF1-Mediated Maintenance DNA Methylation

**DOI:** 10.3390/genes9120600

**Published:** 2018-12-03

**Authors:** Si Xie, Chengmin Qian

**Affiliations:** School of Biomedical Sciences, The University of Hong Kong, Hong Kong, China; h1094160@connect.hku.hk

**Keywords:** DNA methylation, DNMT1, UHRF1, USP7, ubiquitination

## Abstract

Mammalian DNMT1 is mainly responsible for maintenance DNA methylation that is critical in maintaining stem cell pluripotency and controlling lineage specification during early embryonic development. A number of studies have demonstrated that DNMT1 is an auto-inhibited enzyme and its enzymatic activity is allosterically regulated by a number of interacting partners. UHRF1 has previously been reported to regulate DNMT1 in multiple ways, including control of substrate specificity and the proper genome targeting. In this review, we discuss the recent advances in our understanding of the regulation of DNMT1 enzymatic activity by UHRF1 and highlight a number of unresolved questions.

## 1. Introduction

DNA methylation is one of the best-characterized epigenetic changes that have a critical role in numerous biological processes including gene expression, genomic imprinting, X chromosome inactivation and genome stability [1,2,3]. In mammals, DNA methylation predominantly occurs at the C5 position of cytosine in CpG dinucleotides [4,5]. DNA methylation is catalyzed by the DNA methyltransferase family members including DNMT1, DNMT3A and DNMT3B. Another enzymatically inactive member DNMT3L interacts with both DNMT3A and DNMT3B and stimulates their enzymatic activity.

Methylation on CpG sites in promoters generally leads to gene repression by either inhibiting transcription factor binding or recruiting the repressive complex to the promoter region [6]. Therefore, selective methylation on promoter regions of certain genes facilitates the establishment of specific gene expression pattern in differentiated cells. The methylation pattern is tissue-specific and built mainly by de novo DNA methyltransferases DNMT3A and DNMT3B [7,8,9]. Once established, maintenance DNA methyltransferase DNMT1 ensures that the methylation pattern is faithfully propagated throughout successive cell divisions to maintain cell-specific functions in differentiated cells. During the replication process, DNMT1 preferentially catalyzes the conversion of hemi-methylated CpG dinucleotides in daughter strands to fully methylated forms. It is worthy to note that DNMT3A and DNMT3B have been found to be involved in maintaining DNA methylation patterns in specific loci, whereas DNMT1 exhibits de novo DNA methylation under certain circumstances [10,11,12]. In addition, the DNA methylation pattern is dynamic during the developmental processes and to maintain the balance between DNA methylation and demethylation could have a great impact on human health and disease. Similar to other epigenetic modifications, DNA methylation is reversible. 5-Methyl cytosine (5mC) can be further converted to 5-hydroxymethyl cytosine (5hmC), 5-formyl cytosine (5fC) and 5-carboxyl cytosine (5caC) by the DNA methyl-cytosine dioxygenases TET1, TET2 and TET3. It should be pointed out that 5hmC, 5fC and 5caC not just serve as DNA demethylation intermediates but also have distinct regulatory functions in various developmental processes [13,14].

Mounting evidence has demonstrated that DNMT1 alone is not sufficient to maintain the global DNA methylation throughout cell division. Indeed, structural and biochemical analyses have suggested that DNMT1 is a self-inhibited enzyme, as DNMT1 N-terminal regulatory domains including replication foci targeting sequence (RFTS) domain and CXXC domain show autoinhibitory effect on DNMT1 enzymatic activity. Therefore, additional protein factors are required to release DNMT1 from its self-inhibited state. UHRF1 has emerged in recent studies to be one of such regulators [15,16,17]. In this review, we summarize the most recent findings on UHRF1-mediated regulation of DNMT1 recruitment and activation.

## 2. DNMT1 is an Autoinhibited DNA Methyltransferase

**Figure 1:**
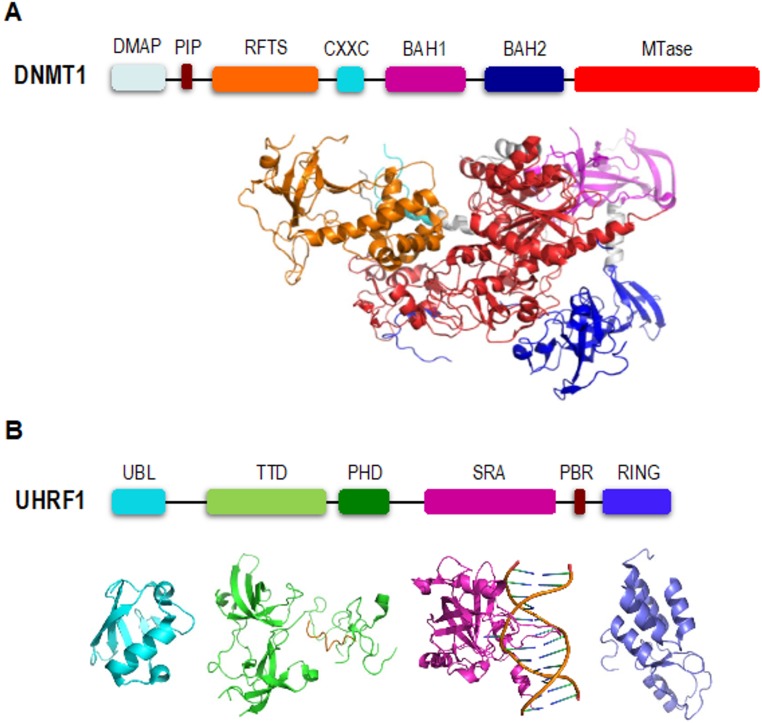
Structures of DNMT1 and UHRF1. (**A**) Domain architecture of DNMT1 and crystal structure of human DNMT1 (aa: 351-1600; PDB: 4WXX) [23]. (**B**) Domain organization of UHRF1 and crystal structures of mouse UHRF1 UBL domain (PDB: 2FAZ), TTD-PHD in complex with H3K9me3 peptide (PDB: 3ASK), SRA in complex with hemi-methylated DNA fragment (PDB: 3CLZ) and UHRF1 RING finger (PDB: 3FL2) [24,25].

DNMT1 is a multi-modular protein consisting of a DMAP1-binding domain, RFTS domain, a CXXC domain, two BAH domains and a C-terminal catalytic domain (Figure 1A). Numerous studies have shown that N-terminal domains of DNMT1 have key regulatory functions. For example, the DMAP1-binding domain and RFTS domain together with a PCNA-interacting protein (PIP) box inserted between them are mainly responsible for DNMT1 stability and its proper localization onto DNA replication site [18,19,20]. At the same time, DNMT1 RFTS domain and CXXC domain can regulate the activity of the catalytic domain. The CXXC domain specifically binds to unmethylated CpG sequences in DNA and positions the CXXC-BAH1 linker between DNA and the catalytic pocket to prevent de novo methylation on those CpG dinucleotides [21,22].

**Figure 2:**
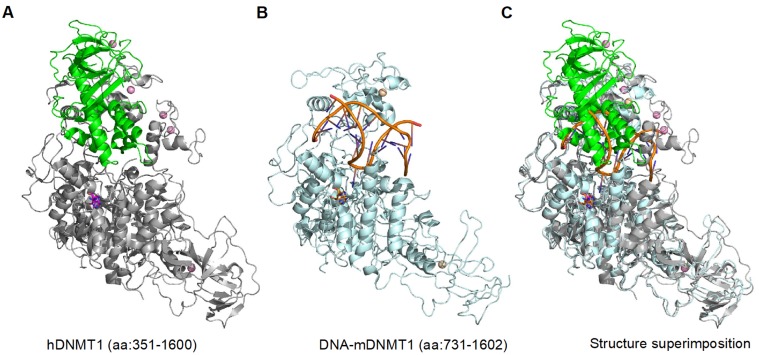
The DNMT1 replication foci targeting sequence (RFTS) domain regulates DNMT1 enzymatic activity through an auto-inhibitory mechanism. (**A**) Crystal structure of human DNMT1 (aa: 351-1600; PDB: 4WXX) in free form [23]. (**B**) Crystal structure of mouse DNMT1 (aa:731-1602; PDB: 4DA4) bound by hemimethylated CpG DNA [31]. (**C**) Superimposition of structures in (**A**) and (**B**) revealed that the RFTS domain occupies DNMT1’s catalytic pocket and prevents substrate DNA binding.

Earlier studies have revealed that RFTS domain regulates DNMT1 enzymatic activity through an auto-inhibitory mechanism, as explained by structural analysis showing that RFTS domain occupies DNMT1 catalytic pocket and prevents substrate DNA binding (Figure 2) [23,26]. Consistent with the structural observation that RFTS domain has intramolecular interaction with the catalytic domain (CD), disruption of RFTS-CD interaction strengthens the RFTS binding to histone H3, which led to abnormal DNMT1 accumulation on chromatin during S-phase [27] and aberrant increase on DNMT1 activity both in vitro and in vivo [28,29]. Such fine-tuned regulatory mechanism of DNMT1 methyltransferase activity could guarantee the faithful inheritance of DNA methylation pattern and maintain tissue-specific functions throughout cell division. Therefore, RFTS has been proposed as a fail-safe lock which protects the genome from aberrant replication-independent DNA methylation [30].

## 3. UHRF1 is Required for Proper Loading of DNMT1 onto Chromatin

UHRF1 is a multifunctional epigenetic regulator that bridges DNA methylation with multiple histone post-translational modifications such as histone methylation, ubiquitination and acetylation. A number of studies have demonstrated that UHRF1 interacts directly with DNMT1 and is essential in DNA methylation maintenance (Figure 1B). The UHRF1 SRA domain specifically binds to hemi-methylated DNA [15,16,25,32,33,34], that helps direct DNMT1 to natural substrate sites. It has also been reported that UHRF1 SRA domain interacts with DNMT1 RFTS domain which could promote the access of DNMT1’s catalytic center to hemi-methylated DNA [29,35]. The UHRF1 PHD finger and tandem tudor domain (TTD) cooperatively recognize H3R2 and H3K9me2/3 mark that may facilitate the proper localization of DNMT1 on genomic loci [24,36,37,38,39,40,41,42,43]. The UHRF1 C-terminal RING finger domain has been shown to function as an E3 ubiquitin ligase responsible for histone H3 ubiquitination [44,45]. Ubiquitinated histone H3 is subsequently recognized by DNMT1 that promotes recruitment of DNMT1 to DNA replication sites [46,47].

## 4. Allosteric Regulation of UHRF1 E3 Ubiquitin Ligase Activity

UHRF1 is proposed to adopt a closed autoinhibited conformation due to extensive inter-domain interactions within the protein [48,49,50]. Getalo et al. reported that a polybasic region (PBR) present between SRA and RING finger of UHRF1 serves as a competitive inhibitor of H3K9me2/3 interaction with the UHRF1 TTD, while the binding of phosphatidylinostiol phosphate (PI5P) to PBR allosterically relieves the inhibition [48]. More recent studies have demonstrated that PBR is a versatile platform that interacts with other partners such as USP7 and DNMT1 [49,51,52]. UHRF1 TTD interacts directly with a histone H3K9-like mimic within DNA ligase 1 (LIG1) [53]. All these interactions can similarly trigger UHRF1 conformational changes and regulate intramolecular activation of UHRF1. On the other hand, the binding of hemi-methylated DNA also causes conformational changes in UHRF1 [49], potentially releases TTD and allosterically regulates UHRF1 E3 ubiquitin ligase activity [50]. Multivalent engagement of hemi-methylated linker DNA and H3K9me2 enhances the enzymatic activity of UHRF1 toward nucleosomal histone substrates [54].

Moreover, UHRF1 UBL domain has just been reported to allosterically regulate UHRF1 E3 ligase activity towards histone H3 [55,56]. UHRF1 UBL and RING finger interact with the ubiquitin conjugating E2 enzyme UbcH5a to stimulate histone H3 ubiquitination.

## 5. UHRF1-Dependent Histone H3 Ubiquitination Stimulates DNMT1 Enzymatic Activity

**Figure 3:**
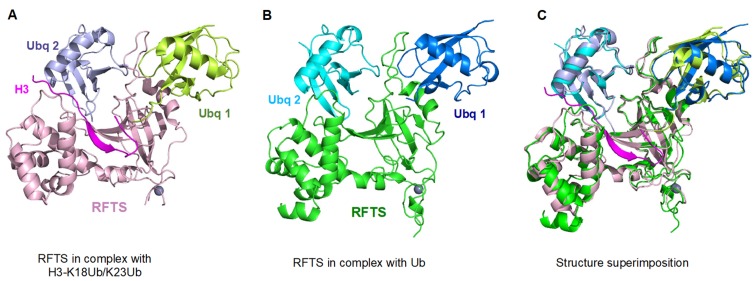
The DNMT1 RFTS domain is a dual mono-ubiquitinated histone H3 binding module. (**A**) Crystal structure of DNMT1 RFTS in complex with ubiquitinated histone H3 (PDB: 5WVO) [57]. (**B**) Crystal structure of DNMT1 RFTS in complex with ubiquitin (PDB: 5YDR) [58]. (**C**) The interaction between RFTS and ubiquitin is essentially the same in these structures. Superimposition of two complex structures gives an RMSD of 0.78Å.

Nishiyama et al. first demonstrated that UHRF1 can ubiquitinate histone H3 on K23, and ubiquitinated H3 is subsequently recognized by DNMT1 [46]. The process actually facilitates the recruitment of DNMT1 to the replication foci. Several studies have further shown that a number of lysine residues including K14, K18 and K23 on histone H3 can be ubiquitinated by UHRF1 [47,50]. Two recent structure-based studies have reported crystal structures of RFTS in complex with ubiquitin and ubiquitinated histone H3 (H3-K18Ub/K23Ub) respectively, which shed light on the molecular mechanism of how UHRF1-dependent H3 ubiquitination regulates DNA methylation maintenance [57,58]. Notably, the RFTS domain of DNMT1 binds ubiquitin with 1:2 stoichiometry in both structures (Figure 3A,B). Superimposition of two complex structures gives a RMSD of 0.78 Å (Figure 3C), consistent with almost the same RFTS/ubiquitin interaction network observed in both structures. Although the ubiquitin-conjugated histone H3 is directly involved in RFTS interaction, it has no obvious effect on ubiquitin recognition by RFTS. This is consistent with the notion in the field that ubiquitin-conjugated substrates usually have no visible impact on the interaction between ubiquitin and ubiquitin-binding domains. RFTS binds to two mono-ubiquitinated histone H3 (H3Ub2) approximately 100 times stronger than histone H3 alone. Intriguingly, RFTS exhibits similar binding affinities to single mono-ubiquitinated histone H3 (H3Ub) and the unmodified H3, suggesting that binding of RFTS to histone H3 and two covalently attached ubiquitin molecules is cooperative [57]. Consistently, mutations on RFTS residues that only participate in the interaction with one of the bound ubiquitin molecules, completely abolish the RFTS association with both ubiquitin molecules, implying that binding of two ubiquitin molecules is interdependent and single mono-ubiquitinated histone H3 is insufficient for DNMT1 recruitment [57,58]. More interestingly, in vitro DNA methylation assays also demonstrate that only two mono-ubiquitinated histone H3, but not ubiquitin alone or unmodified histone H3 could stimulate DNMT1 enzymatic activity [57,58]. However, currently, available structures are unable to explain how exactly the dual mono-ubiquitinated histone H3 alleviates the RFTS auto-inhibitory effect. Further efforts on structural analysis of the complex of two mono-ubiquitinated histone H3 with DNMT1 including RFTS and catalytic domain are required to demonstrate the detailed conformational changes in DNMT1 upon ubiquitinated histone H3 binding.

## 6. DNMT1 Recruitment and Stimulation by UHRF1 N-Terminal UBL Domain

Considering that UHRF1 N-terminal UBL domain structurally resembles ubiquitin, Li et al. went on to test if the UBL domain of UHRF1 could interact with DNMT1 RFTS domain [58]. Indeed, UHRF1 UBL was found to physically interact with DNMT1 RFTS domain. Strikingly, the interaction network of RFTS-UBL is distinct from that of RFTS-ubiquitin although UBL shares high structural similarity with ubiquitin. For example, mutations on RFTS residues that abolished ubiquitin binding showed little or no effect on the interaction with UBL. Unexpectedly, the authors also found that interaction between DNMT1 and UHRF1 UBL could stimulate DNMT1 enzymatic activity. UHRF1 UBL could stimulate the enzymatic activity of DNMT1 segments (aa: 621-1616) and (aa: 351-1616) to a similar level, but H3Ub2 shows no stimulatory effect toward DNMT1(aa: 621-1616), suggesting distinct stimulatory mechanisms by UHRF1 UBL domain and ubiquitinated histone H3 [58]. The authors were able to figure out that UHRF1 UBL not only physically interacts with RFTS but also binds to DNMT1 (aa: 621-1616) and the binding to this DNMT1 fragment is essential for its stimulatory effect on DNMT1 enzymatic activity. It is worthwhile elucidating the detailed molecular basis of the interaction between UHRF1 UBL and DNMT1 in future studies.

UHRF1 UBL has also been revealed to interact with the ubiquitin conjugating E2 enzyme UbcH5a to stabilize the active E2/UHRF1/ubiquitin complex and direct mono-ubiquitination towards histone H3 [55,56], which eventually facilitates the recruitment of DNMT1 to replication foci for efficient DNA methylation maintenance.

## 7. Epigenetic Crosstalk between DNA Methylation and Histone Modifications in Maintenance DNA Methylation

Mounting evidence suggests that crosstalk between DNA methylation and histone modifications occurs to maintain DNA methylation patterns during replication. Previous reports have indicated that binding of UHRF1 to H3K9me2/3 is indispensable for DNMT1 association with chromatin. Disruption of the interaction between UHRF1 and H3K9me2/3 or H3R2 leads to loss of DNMT1 chromatin localization and global hypomethylation [36,41,59]. In contrast, a recent study demonstrated that although disruption of UHRF1 and H3K9me2/3 association leads to hypomethylation, the reduction of DNA methylation occurs globally and does not restrict in the H3K9me2/3 enriched region, suggesting that DNMT1 mediating DNA maintenance methylation is largely independent of H3K9 methylation [59]. Actually, this observation is not surprising considering that the UHRF1 PHD finger but not TTD plays a dominant role in histone H3 binding [24,40,43,60], and expression of UHRF1 with a defective PHD finger failed to restore DNA methylation pattern in UHRF1 knockout cells [47]. The UHRF1 PHD finger recognizes the histone H3 N-terminal tail with unmodified R2. Consequently, methylation of H3R2 by PRMT6 impairs chromatin binding of UHRF1 and induces global DNA hypomethylation [61]. Interestingly, a recent report suggested that side chain guanidinium group of histone H3R8 forms one hydrogen bond with the carbonyl oxygen atom of DNMT1 Tyr564, and forms another hydrogen bond with the side chain of DNMT1 Glu572 [57]. Two previous studies showed that side chain NηH atoms of histone H3R8 form hydrogen bonds with the carbonyl oxygen atom of UHRF1 Asp190 [24,60]. It is likely that methylation on histone H3R8 will antagonize its binding to DNMT1 and UHRF1 based on the above three structure-based analyses.

Histone H3 N-terminal residues such as K14, K18 and K23 can be acetylated or ubiquitinated, acetylation of these residues would preclude ubiquitination on the same sites. Previous studies have shown that HDAC1/2 associate with nascent chromatin during replication [62], DNMT1 and HDAC1/2 reside in a large protein complex [19,63], the presence of HDACs likely makes H3K14, H3K18 and H3K23 sites accessible to UHRF1 for mono-ubiquitination at replicating chromatin of specific genomic loci. On the other hand, acetylation on these lysine residues occurs more frequently at transcriptionally active regions where DNA methylation is rare. Furthermore, a number of studies have shown that DNMT1 and UHRF1 are subject to acetylation, ubiquitination and other post-translational modifications. These post-translational modifications (PTMs) could have an impact on protein stability, modulating protein-protein interactions and even their enzymatic activities, adding another layer of complexity to the regulatory mechanism of maintenance DNA methylation. Future studies are needed to examine if these epigenetic marks are sequentially erased or established during maintenance DNA methylation.

## 8. A Working Model for DNMT1/UHRF1 Complex in Maintenance DNA Methylation

**Figure 4:**
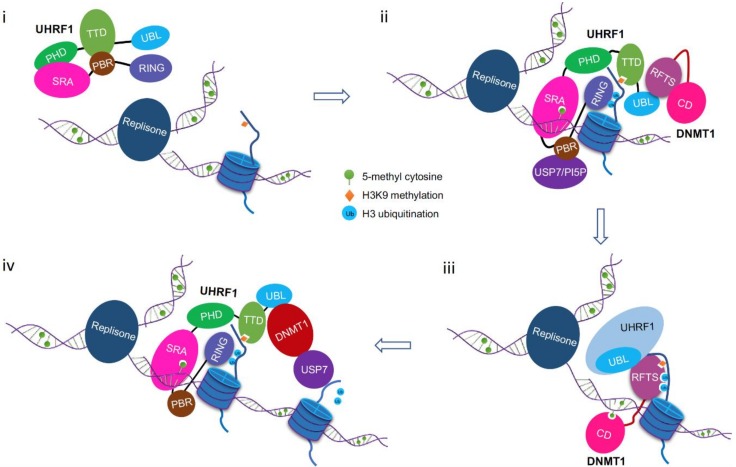
A simplified model for the function of DNMT1/UHRF1 complex in maintenance DNA methylation. (i) UHRF1 adopts a closed conformation in which TTD binds to the PBR motif between SRA and RING finger. (ii) A conformational switch in UHRF1 from inactive closed conformation to active open state is triggered by hemi-methylated DNA binding (and may be facilitated by USP7 or PI5P binding to PBR), which allows TTD-PHD bind to H3K9me2/3 and the RING finger to add ubiquitin to multiple lysine residues of histone H3. (iii) Binding of RFTS to two mono-ubiquitinated histone H3 disrupts the interaction between RFTS and the C-terminal catalytic domain, causing a conformational change in DNMT1 that allows hemi-methylated DNA to access the catalytic center. (iv) After the DNA substrate is fully methylated, DNMT1 is released from the ubiquitinated histone H3 to allow processive DNA methylation along the newly replicated DNA. Again, UHRF1 binds to hemi-methylated DNA first and adds ubiquitin marks to the neighboring histone H3. At the same time, the deubiquitinase USP7 is activated through a currently unknown mechanism to erase ubiquitin marks from histone H3 at methyl DNA fully recovered regions. Likely de-ubiquitination promotes the release of DNMT1 from histone H3 to ensure processive methylation reaction to proceed.

Based on the current knowledge, we propose a simplified working model for the DNMT1/UHRF1 complex in maintenance DNA methylation (Figure 4): (i) The UHRF1 TTD interacts with PBR motif, and the SRA domain binds to the PHD domain. These intramolecular interactions lock UHRF1 in a closed conformation and keep it in an inactive state [48,49,50,51,52]. (ii) During replication, the UHRF1 SRA domain preferentially binds to hemi-methylated DNA, which triggers a conformational change in UHRF1 to dissociate the PHD finger from SRA and release TTD from the PBR motif, allowing TTD-PHD to interact with unmodified H3R2 and H3K9me2/3 marks. This process could be facilitated by the binding of USP7 or PIP5 to PBR. Multivalent engagement of chromatin modifications by various modules of UHRF1 would shift its conformation into an open state in which UHRF1’s E3 ubiquitin ligase activity is greatly stimulated, hence, UHRF1 can effectively add mono-ubiquitin to multiple lysine residues of histone H3. (iii) Through the physical interaction with a number of partner proteins such as PCNA, UHRF1 and ubiquitinated histone H3, DNMT1 is loaded onto chromatin and ready to catalyze methylation on hemi-methylated DNA. The UHRF1-dependent ubiquitination of histone H3 dramatically enhances its interaction with the DNMT1 RFTS domain and displaces the TTD-PHD module of UHRF1 from histone H3. Binding of dual mono-ubiquitinated H3 to DNMT1 will disrupt the intramolecular interaction of RFTS with the C-terminal catalytic region and allow the hemi-methylated DNA substrate to access the catalytic center. (iv) After a hemi-methylated CpG is converted into the fully methylated state, DNMT1 dissociates from the ubiquitinated histone H3 and moves along newly replicated DNA to carry out processive methylation reactions. Again, UHRF1 binds to hemi-methylated DNA first and adds ubiquitin marks to neighboring histone H3. At the same time, the deubiquitinase USP7 is activated to erase ubiquitin moieties from histone H3 at methyl DNA recovered regions [64]. Likely de-ubiquitination promotes the release of DNMT1 from histone H3 to ensure processive methylation reaction to proceed.

## 9. Future Perspective

The past several years have witnessed tremendous advances in our understanding of the regulatory mechanisms involved in maintenance DNA methylation. However, many questions remain to be addressed due to the complex interaction network of DNMT1, UHRF1 and other partner factors. UHRF1-dependent histone H3 ubiquitination is critical for maintenance DNA methylation, but it seems that ubiquitinated histone H3 exists transiently during replication, and USP7 has been suggested to be a histone H3 deubiquitinase [64]. It was previously demonstrated that USP7 promotes the stability of DNMT1 and UHRF1 through preventing two proteins from polyubiquitination and subsequent proteasomal degradation [65,66,67], while the role of USP7 in this process is still under debate. A recent study provides evidence suggesting USP7 is not required for DNMT1 stability and the interaction between USP7 and DNMT1 is unlikely to play a major role in DNMT1 homeostasis [68]. Nonetheless, a number of studies showed that USP7 physically interacts with DNMT1 and UHRF1 [51,66,69]. Binding of USP7 stimulates DNMT1 enzymatic activity in vitro [69]. Moreover, USP7 deubiquitinates the ubiquitinated histone H3 in vitro. Inhibition or depletion of USP7 causes accumulation of ubiquitinated histone H3 and compromises maintenance DNA methylation [64]. Given that the enzymatic activity of USP7 is allosterically regulated through the interaction with other partner proteins [70], it is important to determine how USP7 enzymatic activity is dynamically regulated during the process.

Recent evidence indicates that DNMT1 RFTS domain is a versatile protein interaction module. It has been demonstrated that DNMT1 RFTS domain interacts with the SRA domain and the interaction could stimulate DNMT1 enzymatic activity [29,35,69,71]. More recent studies found that the RFTS domain can interact with histone H3 N-terminal tail [27], but it binds much stronger to two mono-ubiquitinated histone H3 [57]. Given that maintenance DNA methylation is a highly efficient and orchestrated process, so far, more than 20 proteins have been reported to interact with DNMT1 [72]. Therefore, it is likely that some of these proteins, maybe in ubiquitinated form, could interact with RFTS domain to stimulate DNMT1 enzymatic activity during replication.

Lastly, Karg et al. have recently reported PAF15 is a major substrate for mono-ubiquitination by UHRF1 [73]. Dynamic PAF15 ubiquitination has previously been implicated in the regulation of translesion DNA bypass [74]. Interestingly, PAF15 exists in a mono-ubiquitinated form during normal replication, it will be interesting to explore if PAF15 has a role in maintenance DNA methylation.

## References

[1] Smith Z.D., Sindhu C., Meissner A. (2016). Molecular features of cellular reprogramming and development. Nat. Rev. Mol. Cell Biol..

[2] Slotkin R.K., Martienssen R. (2007). Transposable elements and the epigenetic regulation of the genome. Nat. Rev. Genet..

[3] Chang S.C., Tucker T., Thorogood N.P., Brown C.J. (2006). Mechanisms of X-chromosome inactivation. Front. Biosci..

[4] Dodge J.E., Ramsahoye B.H., Wo Z.G., Okano M., Li E. (2002). De novo methylation of MMLV provirus in embryonic stem cells: CpG versus non-CpG methylation. Gene.

[5] Lister R., Pelizzola M., Dowen R.H., Hawkins R.D., Hon G., Tonti-Filippini J., Nery J.R., Lee L., Ye Z., Ngo Q.M. (2009). Human DNA methylomes at base resolution show widespread epigenomic differences. Nature.

[6] Deaton A.M., Bird A. (2011). CpG islands and the regulation of transcription. Genes Dev..

[7] Okano M., Bell D.W., Haber D.A., Li E. (1999). DNA methyltransferases Dnmt3a and Dnmt3b are essential for de novo methylation and mammalian development. Cell.

[8] Kaneda M., Okano M., Hata K., Sado T., Tsujimoto N., Li E., Sasaki H. (2004). Essential role for de novo DNA methyltransferase Dnmt3a in paternal and maternal imprinting. Nature.

[9] Laurent L., Wong E., Li G., Huynh T., Tsirigos A., Ong C.T., Low H.M., Kin Sung K.W., Rigoutsos I., Loring J. (2010). Dynamic changes in the human methylome during differentiation. Genome Res..

[10] Elliott E.N., Sheaffer K.L., Kaestner K.H. (2016). The ‘de novo’ DNA methyltransferase Dnmt3b compensates the Dnmt1-deficient intestinal epithelium. eLife.

[11] Zemach A., McDaniel I.E., Silva P., Zilberman D. (2010). Genome-wide evolutionary analysis of eukaryotic DNA methylation. Science.

[12] Liang G., Chan M.F., Tomigahara Y., Tsai Y.C., Gonzales F.A., Li E., Laird P.W., Jones P.A. (2002). Cooperativity between DNA methyltransferases in the maintenance methylation of repetitive elements. Mol. Cell. Biol..

[13] Ono R., Taki T., Taketani T., Taniwaki M., Kobayashi H., Hayashi Y. (2002). LCX, leukemia-associated protein with a CXXC domain, is fused to MLL in acute myeloid leukemia with trilineage dysplasia having t(10;11)(q22;q23). Cancer Res..

[14] Lorsbach R.B., Moore J., Mathew S., Raimondi S.C., Mukatira S.T., Downing J.R. (2003). TET1, a member of a novel protein family, is fused to MLL in acute myeloid leukemia containing the t(10;11)(q22;q23). Leukemia.

[15] Bostick M., Kim J.K., Esteve P.O., Clark A., Pradhan S., Jacobsen S.E. (2007). UHRF1 plays a role in maintaining DNA methylation in mammalian cells. Science.

[16] Sharif J., Muto M., Takebayashi S., Suetake I., Iwamatsu A., Endo T.A., Shinga J., Mizutani-Koseki Y., Toyoda T., Okamura K. (2007). The SRA protein Np95 mediates epigenetic inheritance by recruiting Dnmt1 to methylated DNA. Nature.

[17] Von Meyenn F., Iurlaro M., Habibi E., Liu N.Q., Salehzadeh-Yazdi A., Santos F., Petrini E., Milagre I., Yu M., Xie Z. (2016). Impairment of DNA methylation maintenance is the main cause of global demethylation in naive embryonic stem cells. Mol. Cell.

[18] Chuang L.S., Ian H.I., Koh T.W., Ng H.H., Xu G., Li B.F. (1997). Human DNA-(cytosine-5) methyltransferase-PCNA complex as a target for p21WAF1. Science.

[19] Rountree M.R., Bachman K.E., Baylin S.B. (2000). DNMT1 binds HDAC2 and a new co-repressor, DMAP1, to form a complex at replication foci. Nat. Genet..

[20] Ding F., Chaillet J.R. (2002). In vivo stabilization of the Dnmt1 (cytosine-5)- methyltransferase protein. Proc. Natl. Acad. Sci. USA.

[21] Pradhan M., Esteve P.O., Chin H.G., Samaranayke M., Kim G.D., Pradhan S. (2008). CXXC domain of human DNMT1 is essential for enzymatic activity. Biochemistry.

[22] Song J., Rechkoblit O., Bestor T.H., Patel D.J. (2011). Structure of DNMT1-DNA complex reveals a role for autoinhibition in maintenance DNA methylation. Science.

[23] Zhang Z.M., Liu S., Lin K., Luo Y., Perry J.J., Wang Y., Song J. (2015). Crystal structure of human DNA methyltransferase 1. J. Mol. Biol..

[24] Arita K., Isogai S., Oda T., Unoki M., Sugita K., Sekiyama N., Kuwata K., Hamamoto R., Tochio H., Sato M. (2012). Recognition of modification status on a histone H3 tail by linked histone reader modules of the epigenetic regulator UHRF1. Proc. Natl. Acad. Sci. USA.

[25] Avvakumov G.V., Walker J.R., Xue S., Li Y., Duan S., Bronner C., Arrowsmith C.H., Dhe-Paganon S. (2008). Structural basis for recognition of hemi-methylated DNA by the SRA domain of human UHRF1. Nature.

[26] Takeshita K., Suetake I., Yamashita E., Suga M., Narita H., Nakagawa A., Tajima S. (2011). Structural insight into maintenance methylation by mouse DNA methyltransferase 1 (Dnmt1). Proc. Natl. Acad. Sci. USA.

[27] Misaki T., Yamaguchi L., Sun J., Orii M., Nishiyama A., Nakanishi M. (2016). The replication foci targeting sequence (RFTS) of DNMT1 functions as a potent histone H3 binding domain regulated by autoinhibition. Biochem. Biophys. Res. Commun..

[28] Bashtrykov P., Rajavelu A., Hackner B., Ragozin S., Carell T., Jeltsch A. (2014). Targeted mutagenesis results in an activation of DNA methyltransferase 1 and confirms an autoinhibitory role of its RFTS domain. ChemBioChem.

[29] Berkyurek A.C., Suetake I., Arita K., Takeshita K., Nakagawa A., Shirakawa M., Tajima S. (2014). The DNA methyltransferase Dnmt1 directly interacts with the SET and RING finger-associated (SRA) domain of the multifunctional protein Uhrf1 to facilitate accession of the catalytic center to hemi-methylated DNA. J. Biol. Chem..

[30] Garvilles R.G., Hasegawa T., Kimura H., Sharif J., Muto M., Koseki H., Takahashi S., Suetake I., Tajima S. (2015). Dual functions of the RFTS domain of Dnmt1 in replication-coupled DNA methylation and in protection of the genome from aberrant methylation. PLoS ONE.

[31] Song J.K., Teplova M., Ishibe-Murakami S., Patel D.J. (2012). Structure-based mechanistic insights into DNMT1-mediated maintenance DNA methylation. Science.

[32] Hashimoto H., Horton J.R., Zhang X., Bostick M., Jacobsen S.E., Cheng X. (2008). The SRA domain of UHRF1 flips 5-methylcytosine out of the DNA helix. Nature.

[33] Arita K., Ariyoshi M., Tochio H., Nakamura Y., Shirakawa M. (2008). Recognition of hemi-methylated DNA by the SRA protein UHRF1 by a base-flipping mechanism. Nature.

[34] Qian C., Li S., Jakoncic J., Zeng L., Walsh M.J., Zhou M.M. (2008). Structure and hemimethylated CpG binding of the SRA domain from human UHRF1. J. Biol. Chem..

[35] Bashtrykov P., Jankevicius G., Jurkowska R.Z., Ragozin S., Jeltsch A. (2014). The UHRF1 protein stimulates the activity and specificity of the maintenance DNA methyltransferase DNMT1 by an allosteric mechanism. J. Biol. Chem..

[36] Nady N., Lemak A., Walker J.R., Avvakumov G.V., Kareta M.S., Achour M., Xue S., Duan S., Allali-Hassani A., Zuo X. (2011). Recognition of multivalent histone states associated with heterochromatin by UHRF1 protein. J. Biol. Chem..

[37] Rajakumara E., Wang Z., Ma H., Hu L., Chen H., Lin Y., Guo R., Wu F., Li H., Lan F. (2011). PHD finger recognition of unmodified histone H3R2 links UHRF1 to regulation of euchromatic gene expression. Mol. Cell.

[38] Hu L., Li Z., Wang P., Lin Y., Xu Y. (2011). Crystal structure of PHD domain of UHRF1 and insights into recognition of unmodified histone H3 arginine residue 2. Cell Res..

[39] Wang C., Shen J., Yang Z., Chen P., Zhao B., Hu W., Lan W., Tong X., Wu H., Li G. (2011). Structural basis for site-specific reading of unmodified R2 of histone H3 tail by UHRF1 PHD finger. Cell Res..

[40] Xie S., Jakoncic J., Qian C. (2012). UHRF1 double tudor domain and the adjacent PHD finger act together to recognize K9me3-containing histone H3 tail. J. Mol. Biol..

[41] Rothbart S.B., Krajewski K., Nady N., Tempel W., Xue S., Badeaux A.I., Barsyte-Lovejoy D., Martinez J.Y., Bedford M.T., Fuchs S.M. (2012). Association of UHRF1 with methylated H3K9 directs the maintenance of DNA methylation. Nat. Struct. Mol. Biol..

[42] Liu X., Gao Q., Li P., Zhao Q., Zhang J., Li J., Koseki H., Wong J. (2013). UHRF1 targets DNMT1 for DNA methylation through cooperative binding of hemi-methylated DNA and methylated H3K9. Nat. Commun..

[43] Rothbart S.B., Dickson B.M., Ong M.S., Krajewski K., Houliston S., Kireev D.B., Arrowsmith C.H., Strahl B.D. (2013). Multivalent histone engagement by the linked tandem Tudor and PHD domains of UHRF1 is required for the epigenetic inheritance of DNA methylation. Genes Dev..

[44] Citterio E., Papait R., Nicassio F., Vecchi M., Gomiero P., Mantovani R., Di Fiore P.P., Bonapace I.M. (2004). Np95 is a histone-binding protein endowed with ubiquitin ligase activity. Mol. Cell. Biol..

[45] Karagianni P., Amazit L., Qin J., Wong J. (2008). ICBP90, a novel methyl K9 H3 binding protein linking protein ubiquitination with heterochromatin formation. Mol. Cell. Biol..

[46] Nishiyama A., Yamaguchi L., Sharif J., Johmura Y., Kawamura T., Nakanishi K., Shimamura S., Arita K., Kodama T., Ishikawa F. (2013). Uhrf1-dependent H3K23 ubiquitylation couples maintenance DNA methylation and replication. Nature.

[47] Qin W., Wolf P., Liu N., Link S., Smets M., La Mastra F., Forne I., Pichler G., Horl D., Fellinger K. (2015). DNA methylation requires a DNMT1 ubiquitin interacting motif (UIM) and histone ubiquitination. Cell Res..

[48] Gelato K.A., Tauber M., Ong M.S., Winter S., Hiragami-Hamada K., Sindlinger J., Lemak A., Bultsma Y., Houliston S., Schwarzer D. (2014). Accessibility of different histone H3-binding domains of UHRF1 is allosterically regulated by phosphatidylinositol 5-phosphate. Mol. Cell.

[49] Fang J., Cheng J., Wang J., Zhang Q., Liu M., Gong R., Wang P., Zhang X., Feng Y., Lan W. (2016). Hemi-methylated DNA opens a closed conformation of UHRF1 to facilitate its histone recognition. Nat. Commun..

[50] Harrison J.S., Cornett E.M., Goldfarb D., DaRosa P.A., Li Z.M., Yan F., Dickson B.M., Guo A.H., Cantu D.V., Kaustov L. (2016). Hemi-methylated DNA regulates DNA methylation inheritance through allosteric activation of H3 ubiquitylation by UHRF1. eLife.

[51] Zhang Z.M., Rothbart S.B., Allison D.F., Cai Q., Harrison J.S., Li L., Wang Y., Strahl B.D., Wang G.G., Song J. (2015). An allosteric interaction links USP7 to deubiquitination and chromatin targeting of UHRF1. Cell Rep..

[52] Gao L., Tan X.F., Zhang S., Wu T., Zhang Z.M., Ai H.W., Song J. (2018). An intramolecular interaction of UHRF1 reveals dual control for Its histone association. Structure.

[53] Ferry L., Fournier A., Tsusaka T., Adelmant G., Shimazu T., Matano S., Kirsh O., Amouroux R., Dohmae N., Suzuki T. (2017). Methylation of DNA ligase 1 by G9a/GLP recruits UHRF1 to replicating DNA and regulates DNA methylation. Mol. Cell.

[54] Vaughan R.M., Dickson B.M., Whelihan M.F., Johnstone A.L., Cornett E.M., Cheek M.A., Ausherman C.A., Cowles M.W., Sun Z.W., Rothbart S.B. (2018). Chromatin structure and its chemical modifications regulate the ubiquitin ligase substrate selectivity of UHRF1. Proc. Natl. Acad. Sci. USA.

[55] DaRosa P.A., Harrison J.S., Zelter A., Davis T.N., Brzovic P., Kuhlman B., Klevit R.E. (2018). A Bifunctional role for the UHRF1 UBL domain in the control of hemi-methylated DNA-dependent histone ubiquitylation. Mol. Cell.

[56] Foster B.M., Stolz P., Mulholland C.B., Montoya A., Kramer H., Bultmann S., Bartke T. (2018). Critical role of the UBL domain in stimulating the E3 ubiquitin ligase activity of UHRF1 toward chromatin. Mol. Cell.

[57] Ishiyama S., Nishiyama A., Saeki Y., Moritsugu K., Morimoto D., Yamaguchi L., Arai N., Matsumura R., Kawakami T., Mishima Y. (2017). Structure of the Dnmt1 reader module complexed with a unique two-mono-ubiquitin mark on histone H3 reveals the basis for DNA methylation maintenance. Mol. Cell.

[58] Li T., Wang L., Du Y., Xie S., Yang X., Lian F., Zhou Z., Qian C. (2018). Structural and mechanistic insights into UHRF1-mediated DNMT1 activation in the maintenance DNA methylation. Nucleic Acids Res..

[59] Zhao Q., Zhang J., Chen R., Wang L., Li B., Cheng H., Duan X., Zhu H., Wei W., Li J. (2016). Dissecting the precise role of H3K9 methylation in crosstalk with DNA maintenance methylation in mammals. Nat. Commun..

[60] Cheng J., Yang Y., Fang J., Xiao J., Zhu T., Chen F., Wang P., Li Z., Yang H., Xu Y. (2013). Structural insight into coordinated recognition of trimethylated histone H3 lysine 9 (H3K9me3) by the plant homeodomain (PHD) and tandem tudor domain (TTD) of UHRF1 (ubiquitin-like, containing PHD and RING finger domains, 1) protein. J. Biol. Chem..

[61] Veland N., Hardikar S., Zhong Y., Gayatri S., Dan J., Strahl B.D., Rothbart S.B., Bedford M.T., Chen T. (2017). The arginine methyltransferase PRMT6 regulates DNA methylation and contributes to global DNA hypomethylation in cancer. Cell Rep..

[62] Sirbu B.M., Couch F.B., Feigerle J.T., Bhaskara S., Hiebert S.W., Cortez D. (2011). Analysis of protein dynamics at active, stalled, and collapsed replication forks. Genes Dev..

[63] Robertson K.D., Ait-Si-Ali S., Yokochi T., Wade P.A., Jones P.L., Wolffe A.P. (2000). DNMT1 forms a complex with Rb, E2F1 and HDAC1 and represses transcription from E2F-responsive promoters. Nat. Genet..

[64] Yamaguchi L., Nishiyama A., Misaki T., Johmura Y., Ueda J., Arita K., Nagao K., Obuse C., Nakanishi M. (2017). Usp7-dependent histone H3 deubiquitylation regulates maintenance of DNA methylation. Sci. Rep..

[65] Du Z., Song J., Wang Y., Zhao Y., Guda K., Yang S., Kao H.Y., Xu Y., Willis J., Markowitz S.D. (2010). DNMT1 stability is regulated by proteins coordinating deubiquitination and acetylation-driven ubiquitination. Sci. Signal..

[66] Cheng J., Yang H., Fang J., Ma L., Gong R., Wang P., Li Z., Xu Y. (2015). Molecular mechanism for USP7-mediated DNMT1 stabilization by acetylation. Nat. Commun..

[67] Ma H., Chen H., Guo X., Wang Z., Sowa M.E., Zheng L., Hu S., Zeng P., Guo R., Diao J. (2012). M phase phosphorylation of the epigenetic regulator UHRF1 regulates its physical association with the deubiquitylase USP7 and stability. Proc. Natl. Acad. Sci. USA.

[68] Yarychkivska O., Tavana O., Gu W., Bestor T.H. (2018). Independent functions of DNMT1 and USP7 at replication foci. Epigenet. Chromatin.

[69] Felle M., Joppien S., Nemeth A., Diermeier S., Thalhammer V., Dobner T., Kremmer E., Kappler R., Langst G. (2011). The USP7/Dnmt1 complex stimulates the DNA methylation activity of Dnmt1 and regulates the stability of UHRF1. Nucleic Acids Res..

[70] Kim R.Q., Sixma T.K. (2017). Regulation of USP7: A high incidence of E3 complexes. J. Mol. Biol..

[71] Achour M., Jacq X., Ronde P., Alhosin M., Charlot C., Chataigneau T., Jeanblanc M., Macaluso M., Giordano A., Hughes A.D. (2008). The interaction of the SRA domain of ICBP90 with a novel domain of DNMT1 is involved in the regulation of VEGF gene expression. Oncogene.

[72] Qin W., Leonhardt H., Pichler G. (2011). Regulation of DNA methyltransferase 1 by interactions and modifications. Nucleus.

[73] Karg E., Smets M., Ryan J., Forne I., Qin W., Mulholland C.B., Kalideris G., Imhof A., Bultmann S., Leonhardt H. (2017). Ubiquitome analysis reveals PCNA-associated factor 15 (PAF15) as a specific ubiquitination target of UHRF1 in embryonic stem cells. J. Mol. Biol..

[74] Povlsen L.K., Beli P., Wagner S.A., Poulsen S.L., Sylvestersen K.B., Poulsen J.W., Nielsen M.L., Bekker-Jensen S., Mailand N., Choudhary C. (2012). Systems-wide analysis of ubiquitylation dynamics reveals a key role for PAF15 ubiquitylation in DNA-damage bypass. Nat. Cell Biol..

